# To live or to die: competitive exclusion of *Schistosoma japonicum* by *Exorchis* sp. in *Oncomelania hupensis*

**DOI:** 10.3389/fimmu.2026.1849466

**Published:** 2026-05-29

**Authors:** Shukun Zhong, Ziyi Dai, Rundong Ji, Huilan Wang, Mao Zheng, Shuaiqin Huang

**Affiliations:** 1 Department of Medical Parasitology, Xiangya School of Basic Medical Sciences, Central South University, Changsha, Hunan, China; 2 Hunan Key Laboratory of Immunology and Transmission Control of Schistosomiasis, Yueyang, Hunan, China; 3 Schistosomiasis Control Institute of Hunan Province (The Third People’s Hospital of Hunan Province), Yueyang, Hunan, China

**Keywords:** competitive exclusion, *Exorchis* sp., immunological mechanisms, *Oncomelania hupensis*, *Schistosoma japonicum*

## Abstract

Schistosomiasis, caused by blood flukes of the genus *Schistosoma*, is a neglected tropical disease whose transmission depends on freshwater snails. *Oncomelania hupensis* is the obligate intermediate host for *Schistosoma japonicum*, disrupting this snail-schistosome relationship is therefore a crucial strategy for disease control and elimination. However, current snail-directed control methods are limited and can adversely affect biodiversity, ecological balance, and environmental health, underscoring the need for deeper mechanistic understanding of factors that naturally regulate schistosome transmission. Notably, prior infection with *Exorchis* sp. has been shown to completely block subsequent infection by *S. japonicum* in *O. hupensis* through within-host competitive exclusion. Therefore, elucidating the biological dynamics and molecular mechanisms underlying this interference competition may thus contribute to the development of novel anti-schistosome strategies. This review systematically synthesizes current knowledge on the life cycle of *Exorchis* sp., the mechanisms driving its competitive dominance over *S. japonicum*, field surveys of its natural distribution, and the underlying immunological mechanisms. By integrating these facets, we aim to advance fundamental understanding of within-host trematode competition and its implications for schistosomiasis transmission ecology. We further discuss how insights into the molecular and immunological mechanisms of this interaction may inform future targeted interventions. Finally, this work presents a unique opportunity to investigate gastropod immunity and host-parasite co-evolutionary dynamics, thereby broadening our knowledge of molluscan immune competence and its role in shaping disease transmission.

## 1 Introduction

Schistosomiasis, a devastating but neglected tropical disease (NTD) caused by parasitic blood flukes of the genus *Schistosoma*, imposes a significant threat to human health and socioeconomic development in tropical and subtropical regions, especially in poor communities with limited access to safe water and sanitation (1, 2). Currently, it affects approximately 250 million people in 78 countries worldwide, with an estimated 800 million people who live in endemic areas at risk of contracting the disease (2, 3). According to the roadmap for NTDs 2021–2030 proposed by the World Health Organization (WHO), schistosomiasis control program goals have shifted from reduction of morbidity to transmission interruption or elimination of schistosomiasis as a public health problem by 2030 in endemic countries (4). Despite continuous global control efforts invested in schistosomiasis prevention and treatment, achievement of this goal still faces several critical problems including the limitation and looming drug resistance of praziquantel (PZQ), environmental toxicity of chemical molluscicides, no available vaccine to prevent reinfection and so on (5–7). Consequently, the development of more effective and eco-friendly alternative anti-schistosome strategies is still necessary.

Infection occurs through contact with fresh water infested with schistosome cercariae, the infective larvae released by the intermediate host snail, thus schistosomiasis is also called as water-borne or snail-borne parasitic disease (8). Meanwhile, snail control is recognized as a crucial measure to schistosomiasis elimination. Snails can be controlled by chemical control using molluscicides (synthetic or natural origin), physical control using environmental modification, and biological control using predators of aquatic snails (9–11). Over the past few decades, snail control plays a pivotal role in the comprehensive prevention and control measures of schistosomiasis (12, 13). However, snail-directed control methods are limited and have potential negative impacts on biodiversity, ecological balance, and environment protection (9–13). Therefore, a deeper mechanistic understanding of factors that naturally regulate schistosome transmission may inform the development of ecologically informed control strategies, especially under low-prevalence conditions of schistosomiasis.

The amphibious freshwater snail *Oncomelania hupensis* is the obligate intermediate host of *Schistosoma japonicum*, which is the prevalent species with distribution in regions along the Yangtze River basin in China, across the islands of the Philippines, and a small focus of Indonesia (12, 13). Additionally, it has been clearly identified as the first intermediate host for *Exorchis* sp. distributed in the basins of Dongting and Poyang Lakes, a trematode that uses the carnivorous fish *Silurus asotus* as its definitive host, but not humans (14, 15). Typically, it has been well confirmed an individual snail in areas endemic for trematodes serves as host to harbor only one species of larval trematode simultaneously (16). This observation leads to the hypothesis that in snails co-infected with *S. japonicum* and *Exorchis* sp., one species will competitively exclude the other, raising the question of which parasite will ultimately prevail in the co-infection. Through extensive experimental verification, Tang et al. demonstrated that prior infection with *Exorchis* sp. can completely block and inhibit subsequent infection by *S. japonicum* in *O. hupensis* (16). This finding reveals a potent within-host competitive exclusion mechanism that, in principle, could disrupt the parasite’s transmission route to humans without the use of chemical molluscicides or anthelmintics. However, it is critical to acknowledge that translating this naturally occurring competitive interaction into a practical control strategy faces considerable ecological challenges. Specifically, *Exorchis* sp. is parasitic to fish and may utilize a broad range of snail intermediate hosts, raising legitimate concerns regarding non-target effects and aquatic ecosystem health. Although these ecological barriers currently preclude the direct environmental application of *Exorchis* sp. as a targeted biological control agent, elucidating the molecular mechanisms underlying this interference competition could contribute to the discovery of novel immune effectors and anti-schistosomal drug targets.

In this review, we synthesize current research on the competitive interaction between *Exorchis* sp. and *S. japonicum* within *O. hupensis* and explore the immune mechanisms underlying their within-host competitive exclusion. This review aims to deepen our understanding of the biological dynamics governing trematode competition and to illuminate how these mechanistic insights may inform future schistosomiasis control strategies.

## 2 *O. hupensis* serves as the common intermediate host of *Exorchis* sp. and *S. japonicum*

Schistosomes are digenetic trematodes that require humans or other mammals as definitive hosts for sexual reproduction and gastropod snails as intermediate hosts for asexual reproduction (17). *O. hupensis*, as the unique intermediate host of *S. japonicum*, provides a suitable environment for the parasite’s amplification and development (17, 18). The life cycle of *S. japonicum* commences when eggs excreted in definitive host feces hatch upon immersion into freshwater, releasing free-swimming short-lived ciliated miracidia. These highly motile miracidia seek out and penetrate the intermediate host snail, *O. hupensis*, where asexual reproduction begins. Inside the snail, each miracidium transforms into a mother sporocyst, which gives rise to numerous daughter sporocysts. Within these daughter sporocysts, thousands of cercariae develop and are periodically released into the water. Upon contact with infested water, cercariae rapidly penetrate the skin of a definitive host including human, buffalo or dog, migrate through the systemic circulation to the hepatic portal vessels, and mature into adult worms. Following male-female pairing, the adults migrate against the blood flow in the mesenteric vessels of the intestine to reside and reproduce sexually. The cycle is completed when eggs produced by the females are excreted in the host’s feces back into the environment (**Supplementary Figure 1**).

As the cryptogonimid trematodes, *Exorchis* sp. have an obligate, complex three-host life cycle: siluriform fish serve as definitive hosts, mollusks as first intermediate hosts, and various cyprinid fish as second intermediate hosts (14, 15, 19). Within the genus *Exorchis*, the seven valid species are broadly categorized by size into ‘large’ worms (*E. oviformis* and *E. macrobursae*) and ‘small’ worms (*E. convictus* sp. n., *E. multivitellarius*, *E. ovariolobularis*, *E. dongtinghuensis*, and *E. mupingensis*) (19–21). Notably, the latter three species are capable of utilizing *O. hupensis* as their first intermediate host. However, current evidence indicates that host specificity varies among *Exorchis* species. Specifically, experimental and field studies have confirmed that *E. ovariolobularis* can utilize *Stenothyra toucheana* as a competent intermediate host in the wild (22), completing its full larval development from miracidium to mature cercaria in the absence of *O. hupensis*. Although no alternative intermediate hosts have been documented for the other two species to date, this finding demonstrates that host plasticity exists within the genus and that some *Exorchis* species may exploit a broader range of snail intermediate hosts in natural settings.

The snail acquires infection by ingesting mature *Exorchis* sp. eggs that have been released into the aquatic environment via the feces of the infected definitive host, *S. asotus*. Within the snail, the parasite undergoes sequential larval development through the stages of germinal cells, rediae, and cercariae. At a temperature range of 25-35 °C, this intramolluscan phase lasts approximately seven months, culminating in the emergence of mature cercariae. These cercariae then seek out a second intermediate host (typically a small cyprinid fish) to penetrate it, and encyst as metacercariae in its tissues. Upon consumption of these infected cyprinid fish, *S. asotus* becomes infected. The parasites develop into mature adults in the intestinal tract, from which eggs are subsequently released in the feces, thereby restarting the life cycle (**Supplementary Figure 1**).

## 3 *Exorchis* sp. as a natural competitor of *S. japonicum* in *O. hupensis*

**Figure 1:**
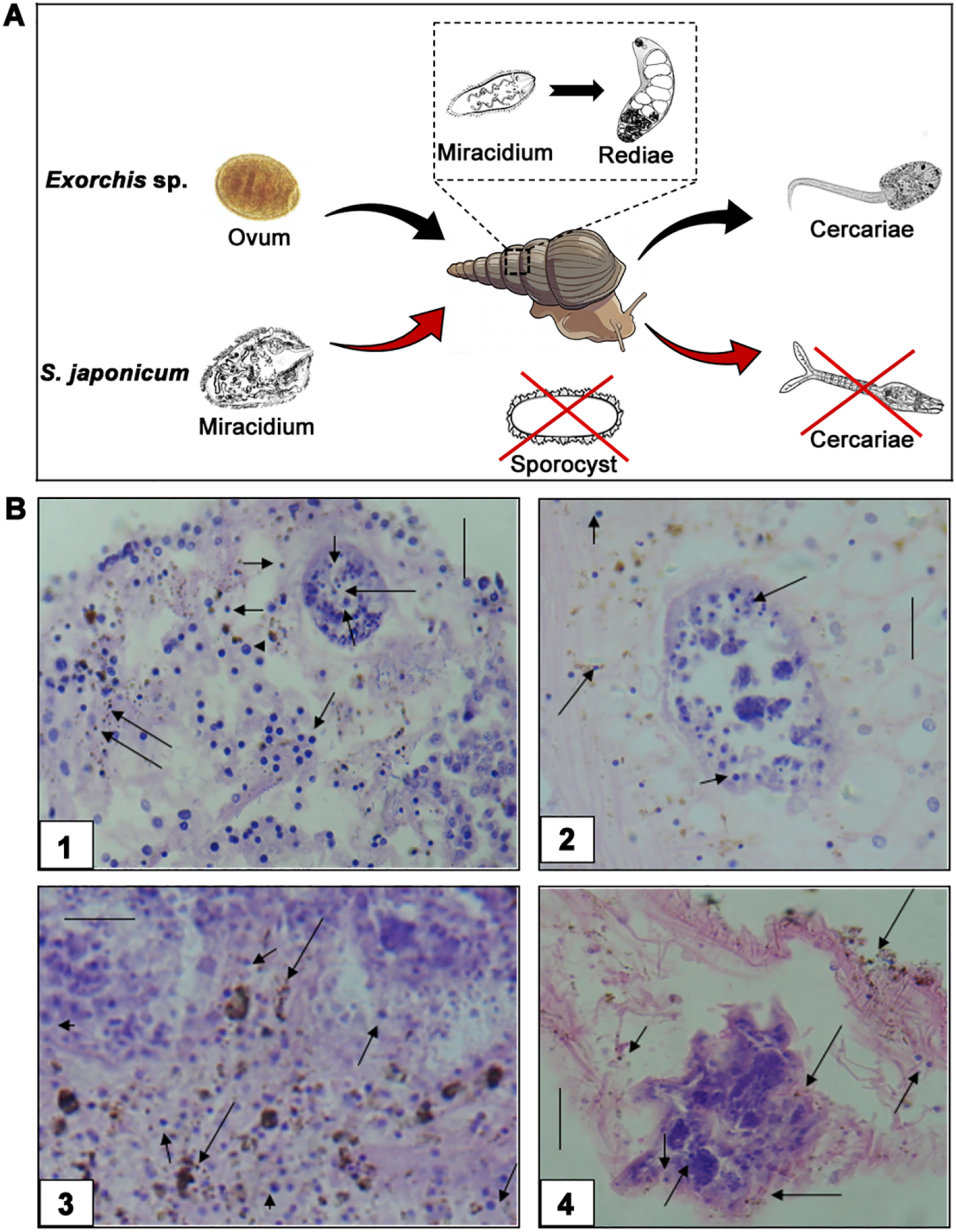
*Exorchis* sp. competitively antagonizes the parasitism and survival of *Schistosoma japonicum* within the snail host *Oncomelania hupensis*. **(A)** Schematic diagram of the antagonistic effect of *Exorchis* sp. against *S. japonicum* in *O. hupensis*. **(B)** Development of *S. japonicum* larvae blocked in *O. hupensis* by prior infection with *Exorchis* sp. larvae. 1: Following initial infection with *Exorchis* sp. and subsequent challenge with *S. japonicum*, hemocytes undergo marked proliferation and migrate toward the schistosome sporocyst. 2: Infiltration by numerous large and medium hemocytes into abnormal 39-day-old mother sporocysts was observed, accompanied by abundant golden-yellow granular secretions. 3: Abundant black granular secretions surrounded the 82-day-old abnormal mother sporocysts. 4: The schistosome mother sporocyst disintegrated and died. The images are derived from the study published by Tang et al. (26).

In the complex life cycle of digenean trematodes, the snail is an indispensable obligate host for generating cercariae that infect humans (9–11). Thus, during the long-term co-evolution, successful infection of an individual snail is primarily determined by the competitive balance between schistosome infectivity and snail immune resistance. Once this equilibrium is disrupted, the parasitic relationship between the two will be compromised, thereby blocking the transmission route of schistosomiasis. Since *Exorchis* sp. can also utilize *O. hupensis* as an intermediate host, this raises the hypothesis that it may interfere with or break the obligate parasitic balance between the snail and *S. japonicum* (**Figure 1A**).

Research initially revealed that *Exorchis* sp. infection provokes a markedly stronger immune response in *O. hupensis* than *S. japonicum* infection, characterized by a substantial increase in hemocyte numbers and secretion volume (23, 24). Critically, subsequent pre-infection with *Exorchis* sp. completely blocks and destroys the developing *S. japonicum* larvae, with these degenerating forms often surrounded and infiltrated by elevated levels of hemocytes and secretions (**Figure 1B**) (16). Further investigation indicated that the antagonistic effect on *S. japonicum* larvae in dually infected snails intensifies with longer co-infection intervals (25). Concurrently, a shift in the hemocyte population occurs, marked by a decline in both total numbers and the proportions across different size classes as co-infection progresses (26, 27). Ultimately, the secretions and the abnormal larvae themselves adopt a more complex and dense morphology (28, 29). These findings demonstrate that *Exorchis* sp. can competitively antagonize the survival and development of *S. japonicum* within the snail host, revealing a potent within-host competitive exclusion mechanism. However, key translational questions remain unanswered, including the magnitude of transmission reduction achievable under natural conditions and the ecological risks posed by the parasite’s broad host range and potential fish pathogenicity. Systematic field surveys and deeper mechanistic studies are therefore needed to address these uncertainties.

More than 90% of *Oncomelania* snails inhabit the areas around Dongting Lake, Poyang Lake, and the beaches of the Yangtze River, which coincide with the current primary endemic foci of schistosomiasis japonica (9, 10). It is therefore imperative to conduct field surveys in these key endemic zones to assess the natural distribution and prevalence of *Exorchis* sp. and to evaluate its ecological interactions with *O. hupensis* under field conditions. Field survey results from the Muping Lake area in the Dongting Lake basin (Tang et al.) indicated a nearly 3.3% prevalence of *Exorchis* sp. in *O. hupensis* snails versus 99.31% in *S. asotus*, with the latter harboring an average of 115.4 worms per fish (23). Moreover, a field survey conducted from 2012 to 2016 in the marshlands of Poyang Lake revealed an *Exorchis* sp. infection rate of over half (65.79%) in *S. asotus*, with an average intensity of 14.21 worms per fish; meanwhile, the average infection rate in *O. hupensis* snails was 1.11% (30). The presence of abundant *Exorchis* sp. populations in these areas provides a valuable opportunity to investigate the ecological and immunological dynamics of within-host trematode competition in natural settings. Moreover, the marked discrepancy between low infection prevalence in a single snail species and high prevalence in the definitive fish host represents a recognized ecological pattern that often signals the utilization of a broader range of intermediate snail hosts in the wild.

## 4 Potential mechanisms of antagonism against *S. japonicum* by *Exorchis* sp. in *O. hupensis*

Mollusks, including snails, defend against pathogens mainly via an innate immune system of immune cells, effector molecules, and cytokines. Snail hemocytes are pivotal, executing functions like phagocytosis, encapsulation, and coagulation, and producing cytotoxic agents including nitric oxide (NOS) and reactive oxygen species (ROS) (31–33). In the co-infected *O. hupensis* model, *Exorchis* sp. infection triggers a cascade of immune responses: inducing hemocyte proliferation, recruiting them to sites of *S. japonicum* larvae, and stimulating the production of effectors, thereby mounting an effective attack against the invading parasites (16). One potential mechanism is that *Exorchis* sp. may outcompete *S. japonicum* within *O. hupensis* by establishing a dominant interaction that disrupts the host-parasite balance. This shifts the immune equilibrium in favor of the snail, leading to the blockage and destruction of *S. japonicum* larvae. A second hypothesis involves trained innate immunity in *O. hupensis*, wherein prior exposure to *Exorchis* sp. antigens primes the snail’s immune system (34, 35). This priming leads to a significantly stronger response upon subsequent challenge with *S. japonicum* miracidia, enhancing parasite clearance. Alternatively, a third hypothesis introduces the possibility that the underlying mechanism is a distinct immune strategy in invertebrates, differing fundamentally from vertebrate innate immunity. However, these three hypotheses require further exploration and verification.

An integrated transcriptomic and iTRAQ-based proteomic analysis has identified several critical differentially expressed proteins associated with the antagonistic effect of *Exorchis* sp. against *S. japonicum* in *O. hupensis*, including the macrophage migration inhibitory factor (OhMIF) and thioredoxin-related protein of 14 kDa (OhTRP14) (36). Later investigation demonstrated that OhMIF knockdown significantly diminished key hemocyte subpopulations, specifically phagocytic cells and those with larger volume and higher granularity in circulating hemolymph. These findings suggest OhMIF’s essential roles in promoting hemocyte activation, differentiation, and their targeted recruitment to infection sites, thereby facilitating an effective immune response (37–39). Furthermore, it was revealed that upon schistosome infection, snail OhTRP14 expression is markedly upregulated, accompanied by a significant increase in ROS levels within circulating hemocytes. In contrast, knockdown of OhTRP14 results in an even greater elevation of hemocytic ROS in infected snails (40, 41). This indicates that OhTRP14 likely plays a crucial role in regulating the oxidative stress response during infection. In addition, studies indicated that TLRs are involved in the innate immune response of *O. hupensis* against early *S. japonicum* infection, and may further regulate the activation of distinct hemocyte populations (42). Given this role, OhMyD88, a downstream adaptor in the TLR signaling pathway, is also closely associated with these dynamic changes (43).

**Figure 2:**
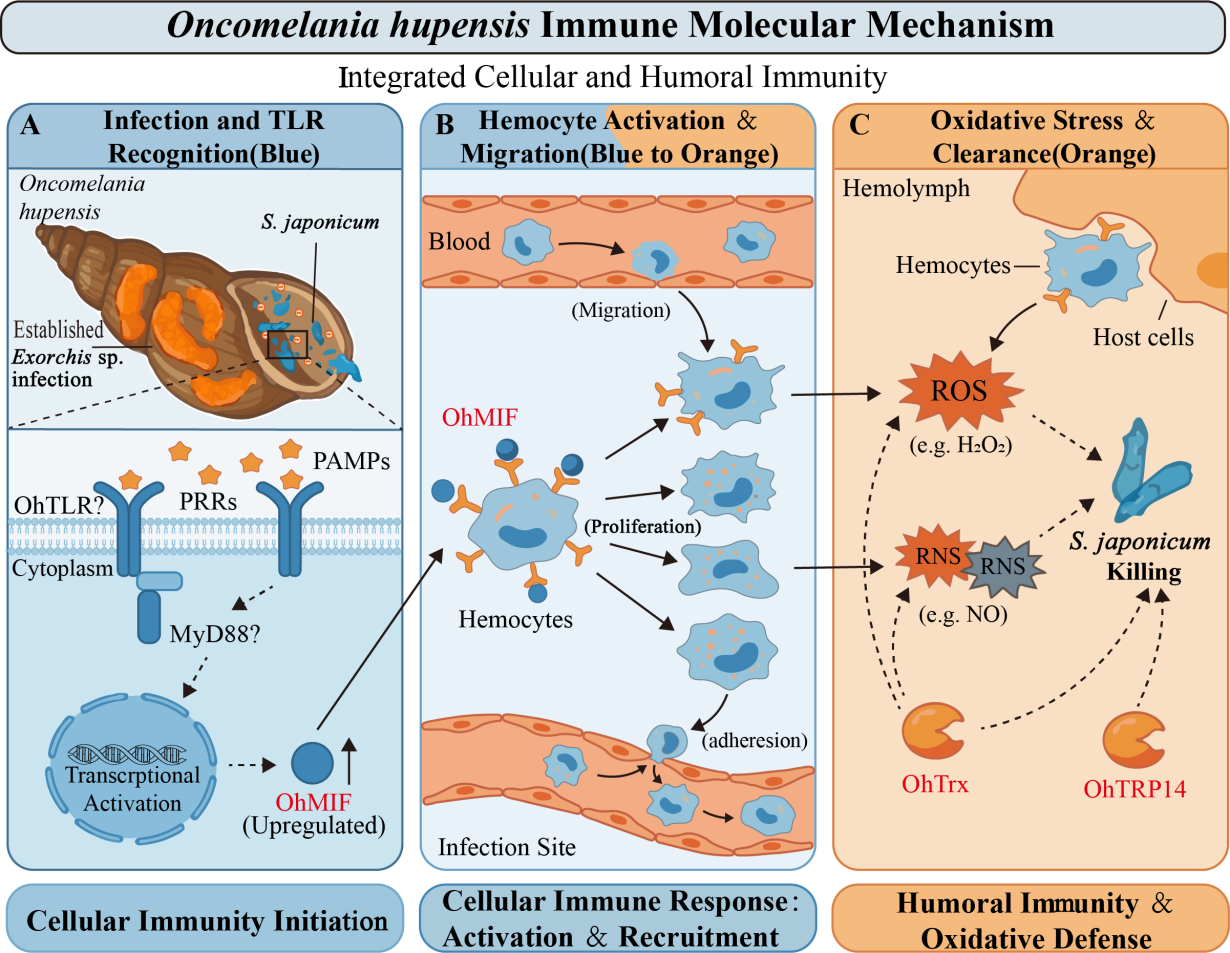
Potential mechanisms of antagonism against *S. japonicum* by *Exorchis* sp. in *O. hupensis*. Activation of the TLR/MyD88 pathway by *Exorchis* sp. infection triggers OhMIF-mediated hemocyte proliferation and migration. Recruited hemocytes then enhance ROS-related metabolism, releasing cytotoxic effectors (NOS/ROS) that attack schistosome larvae. It could be divided into three processes: Cellular immunity initiation, Cellular immune response, and Humoral immunity & Oxidative defense. **(A)** Infection and TLR recognition (Blue). **(B)** Hemocyte activation & migration (Blue to Orange). **(C)** Oxidative stress & clearance (Orange).

Collectively, infection by *Exorchis* sp. may significantly activate the TLR/MyD88 signaling pathway in *O. hupensis*, enabling antigen recognition and the production of inflammatory cytokines/chemokines such as OhMIF. This response in turn promotes hemocyte proliferation and their migration to infection sites. Subsequently, the recruited phagocytic hemocytes activate pathways involved in ROS-related metabolism, leading to the production and release of cytotoxic agents (e.g., NOS and ROS) to mount a direct attack against the schistosome larvae (**Figure 2**).

## 5 Conclusions

This article presents a comprehensive overview of the competitive interaction between *Exorchis* sp. and *S. japonicum* within their shared snail host and examines its implications for schistosomiasis transmission ecology. We summarize current knowledge on the life cycle of *Exorchis* sp., the phenotypic outcomes of within-host competition, field surveys of its natural distribution, and the potential immunological mechanisms underlying competitive exclusion. By integrating these facets, we aim to advance the fundamental understanding of trematode interference competition and to critically assess how these mechanistic insights may inform the development of ecologically sound strategies for schistosomiasis control.
