# Supplementary material for: To live or to die: competitive exclusion of Schistosoma japonicum by Exorchis sp. in Oncomelania hupensis
Source: Front Immunol. 2026 May 29;17:1849466. doi: 10.3389/fimmu.2026.1849466 (PMC13267002; doi:10.3389/fimmu.2026.1849466)
Supplement: Supplementary file 1 [file DataSheet1.docx]

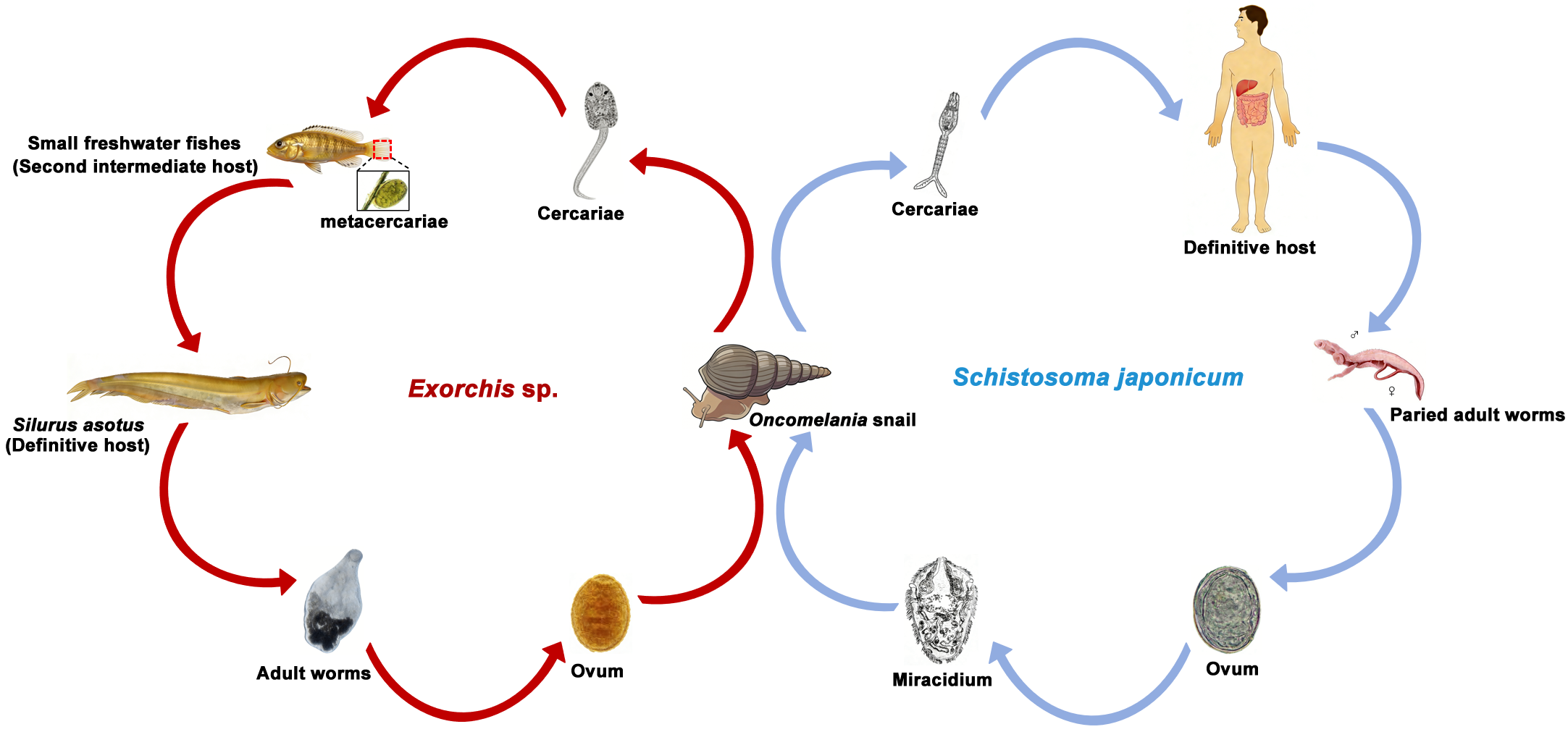


**Supplementary Fig. 1. Life cycles of *Exorchis* sp. and *Schistosoma japonicum*.** *Exorchis* sp. completes its life cycle via two intermediate hosts and one definitive host. Adults in the intestine of *S. asotus* release eggs via feces; after ingestion by *O. hupensis* snails, the parasite undergoes larval development and produces cercariae, which then infect cyprinid fish and encyst as metacercariae, ultimately transmitting to *S. asotus* upon predation. *S. japonicum* begins its life cycle when eggs from definitive host feces hatch in freshwater, releasing miracidia that infect the intermediate snail host *O. hupensis*. After asexual reproduction within the snail, thousands of cercariae are released and penetrate the skin of definitive hosts (e.g., humans, buffaloes), maturing into adult worms that pair and produce eggs, which are excreted in feces to restart the cycle.
